# From Genome to Drugs: New Approaches in Antimicrobial Discovery

**DOI:** 10.3389/fphar.2021.647060

**Published:** 2021-06-09

**Authors:** Federico Serral, Florencia A. Castello, Ezequiel J. Sosa, Agustín M. Pardo, Miranda Clara Palumbo, Carlos Modenutti, María Mercedes Palomino, Alberto Lazarowski, Jerónimo Auzmendi, Pablo Ivan P. Ramos, Marisa F. Nicolás, Adrián G. Turjanski, Marcelo A. Martí, Darío Fernández Do Porto

**Affiliations:** ^1^ Instituto de Cálculo, Facultad de Ciencias Exactas y Naturales, Universidad de Buenos Aires, Buenos Aires, Argentina; ^2^ Departamento de Química Biológica, Facultad de Ciencias Exactas y Naturales, Universidad de Buenos Aires, Buenos Aires, Argentina; ^3^ Instituto de Química Biológica de la Facultad de Ciencias Exactas y Naturales (IQUIBICEN) CONICET, Ciudad Universitaria, Buenos Aires, Argentina; ^4^ Departamento de Bioquímica Clínica, Instituto de Investigaciones en Fisiopatología y Bioquímica Clínica (INFIBIOC), Facultad de Farmacia y Bioquímica, Universidad de Buenos Aires, Buenos Aires, Argentina; ^5^ Consejo Nacional de Investigaciones Científicas y Técnicas (CONICET), Buenos Aires, Argentina; ^6^ Centro de Integração de Dados e Conhecimentos para Saúde (CIDACS), Instituto Gonçalo Moniz, Fundação Oswaldo Cruz (FIOCRUZ), Salvador, Brazil; ^7^ Laboratório Nacional de Computação Científica (LNCC), Petrópolis, Brazil

**Keywords:** drug discovery, drug target, metabolic reconstruction, structural modeling, target prioritization, virtual screening

## Abstract

Decades of successful use of antibiotics is currently challenged by the emergence of increasingly resistant bacterial strains. Novel drugs are urgently required but, in a scenario where private investment in the development of new antimicrobials is declining, efforts to combat drug-resistant infections become a worldwide public health problem. Reasons behind unsuccessful new antimicrobial development projects range from inadequate selection of the molecular targets to a lack of innovation. In this context, increasingly available omics data for multiple pathogens has created new drug discovery and development opportunities to fight infectious diseases. Identification of an appropriate molecular target is currently accepted as a critical step of the drug discovery process. Here, we review how diverse layers of multi-omics data in conjunction with structural/functional analysis and systems biology can be used to prioritize the best candidate proteins. Once the target is selected, virtual screening can be used as a robust methodology to explore molecular scaffolds that could act as inhibitors, guiding the development of new drug lead compounds. This review focuses on how the advent of omics and the development and application of bioinformatics strategies conduct a “big-data era” that improves target selection and lead compound identification in a cost-effective and shortened timeline.

## Introduction

Antibiotics have revolutionized medicine in many aspects, and countless lives have been saved since their discovery at the beginning of the 20th century. However, although antimicrobials have enabled the control of most bacterial diseases considered deadly in the pre-antibiotic era, the emergence of resistant or multiresistant strains, often called "superbugs", is now a huge source of concern for human health. The extraordinary genetic capacities of bacteria have benefited from man’s overuse of antibiotics, leading to multiple antibiotic resistance mechanisms for each antibiotic introduced in the clinical practice (Davies and Davies, 2010). In this context, novel drugs or therapies are urgently required. Still, in a scenario where private investment in the development of new antimicrobials is declining, efforts to combat drug-resistant infections is becoming a worldwide concern.

Generally, antibiotic discovery and development processes are ineffective and costly. It is predicted that around 90% of drugs entering phase 1 clinical trials will not reach approval and that the overall cost for each approved compound is about 1.4 billion dollars (Hay et al., 2014; DiMasi et al., 2016). The decision-making process in a drug discovery project requires a thorough understanding of as many variables as possible to maximize the chance of success. The reasons for the failure of many new antimicrobial development projects range from inadequate selection of the molecular targets to a lack of innovation and, quite significantly, the appearance of severe side effects. However, the availability of pathogen genomic-scale datasets has created new opportunities for drug discovery, including those against new resistant and multiresistant strains. Subtractive genomics, structural bioinformatics, and metabolic pathways analysis approaches are currently applied for the development of new drugs and fight antimicrobial resistance, acting as a complement to traditional wet-lab approaches. Although not enough time has elapsed to exploit all capabilities of in silico approaches in drug discovery, target-based drug discovery has been effective for many therapeutic targets, most notably for HIV/AIDS (Zhan et al., 2016), and was also successful in identifying potent antibacterial inhibitors of peptide deformylase (Hackbarth et al., 2002). Other examples of genomic approaches that resulted in promising compounds include AFN-1252, a selective inhibitor of the *Staphylococcus aureus* enzyme enoyl-acyl carrier protein reductase, FabI, which showed potent *in vitro* activity and *in vivo* efficacy (Kaplan et al., 2012). Early genome-wide studies pointed to the essentiality of proteins involved in fatty acid biosynthesis that, coupled to structural differences between enzymes from bacteria and mammals, made these a noteworthy target (Forsyth et al., 2002). BamA, a component of the β-barrel assembly machine of Gram-negative bacteria, has also been proposed as a target due to its essentiality and extensive conservation in these organisms. A monoclonal antibody that selectively inhibits this protein has been developed and demonstrated to have bactericidal activity (Storek et al., 2018). Other inhibitors targeting Gram-negative outer membrane proteins have also been proposed (MacNair et al., 2020). Combined, these examples reinforce the utility of target-based approaches that, informed by genome evidence, can result in the successful identification of novel drug candidates.

Targeted drug development projects consist of several steps that range from candidate selection and validation, the performance of *in vitro* and *in vivo* experiments to identify lead and candidate molecules, pre-clinical development in animal models, and finally, clinical trials in human subjects to establish safety and effectiveness. Along this long and winding road, several significant challenges must be met to avoid failure, and as in any race, an optimal start is of great advantage. The mentioned advent of omics approaches (e.g., genomics, transcriptomics, and proteomics) has fostered the development of bioinformatics tools guiding to a “big-data era” that allows improved identification of putative targets and lead compounds. Other informatics approaches to enhance antimicrobial discovery, such as Machine Learning (ML), are out of the scope of the present work and are reviewed elsewhere (Lau et al., 2021). Opportunities to apply ML occur in all stages of antimicrobial discovery (Vamathevan et al., 2019; Lau et al., 2021). Examples include target validation, identification of prognostic biomarkers, and analysis of digital pathology data in clinical trials. Halicin is one of the most notable discoveries of new antimicrobials using ML techniques (Stokes et al., 2020). This drug was effective against many multidrug resistant microbes *in vitro* and *in vivo*.

In this review, we will focus on the different bioinformatics strategies used for prioritizing drug targets in pathogens. Particularly, we include results of prioritized targets with their potential molecule inhibitor candidates for two bacteria that cause endemic diseases in Latin American countries, namely *Mycobacterium tuberculosis* (Mtb) and *Bartonella bacilliformis* (Bb)*.*

## How to Prioritize Drug Targets in Pathogenic Bacteria?

Since experimental research of putative drug targets is time-consuming and expensive, it is worthwhile to conduct bioinformatic analysis to select proteins that are good candidates as molecular targets for antimicrobial discovery projects. These analyses consider features commonly thought to be desirable in a target, including druggability (whether drug-like compounds are likely to interact with the protein), essentiality (which suggest that inhibiting the target function will have the desired bactericidal effect), specificity/selectivity (potential for inhibiting the pathogen without harming the host and its microbiota), and relevance in metabolic stages of the pathogen during infection.

From a general point of view, druggability is a concept used to describe the ability of a given protein to bind a drug-like molecule, which in turn modulates its function in some “desired” way (Gashaw et al., 2012). From a structural point of view, it can be related to the likelihood that a small molecule binds a given protein target with high affinity (Sheridan et al., 2010), a property usually referred to bindability. Taking this into account, druggable proteins should have a well-defined pocket with suitable physicochemical properties to bind a drug. Our group has developed a fast whole genome approach for druggability prediction based on the open-source algorithm fpocket (http://fpocket.sourceforge.net/) (Guilloux et al., 2009), which combines several physicochemical descriptors to estimate the druggability of the pockets present in proteins. This approach was extensively tested, both on experimental structures and homology-based models, in the context of whole proteome target search studies in our previous works on the subject (Radusky et al., 2014; Defelipe et al., 2016; Ramos et al., 2018; Sosa et al., 2018; Farfán-López et al., 2020). Based on previous analysis of the druggability score distribution, for all pockets that host a drug-like compound in the Protein Data Bank (Sussman et al., 1998), we have classified pockets into four categories: non-druggable (ND), poorly druggable (PD), druggable (D), and highly druggable (HD). Good candidate targets are, from a structural standpoint, proteins that fall either into D or HD classes. Most of the pockets that actually host a drug in the PDB (80%) could be classified as druggable or highly druggable by our methodology.

Moving from the structural to a more general druggability concept, the early steps of rational antimicrobial target identification usually involve integrating the structural druggability assessment with the information present in the host and pathogen whole genomes. This strategy, called subtractive genomics, allows to select those targets relevant for the pathogen and absent in the host. Identifying a group of proteins that are essential to pathogens but are not present in the host minimizes the chance of unwanted side effects during treatment (Barh et al., 2011). Three hierarchical levels (sequence → DNA/protein, structure → protein, and enzymatic/regulatory reactions → regulatory/metabolic network) have generally been used alone to select candidate targets (Radusky et al., 2015; Defelipe et al., 2016; Kaur et al., 2017; Wadood et al., 2017; Uddin and Jamil, 2018). We drive our focus to the analysis of these multiple omics layers under an integrative framework.

### Metabolic Reconstruction Contextualizes Target Importance and Directs Selection in Early Phases

The first layer of information that can be used to direct target prioritization efforts is the evaluation of the metabolic importance of a given protein. Metabolism refers to the set of biochemical reactions and regulatory pathways leading to cellular homeostasis and functioning. Early studies on microbial metabolism elucidated the major pathways related to energy production, amino acid synthesis, and lipid formation, which, combined with the current availability of full genomes and proteomes, helped set the stage for the study of metabolism on a large-scale. Computational methods that rely on whole-genome sequences, gene annotations, or both, allow for rapid generation of an initial metabolic draft for any given organism, which must be followed by careful manual curation to achieve a high-quality metabolic reconstruction. Pathway Tools (Karp et al., 2015) is one of such software providing a module (PathoLogic) that takes as input the genome and associated annotations of an organism of interest and, by mapping these annotations onto enzymatic reactions within the MetaCyc database using an enzyme-name matching tool, infers the set of reactions (or the reactome) for the desired species. A pathway-scoring algorithm is employed to predict pathways within the expected taxonomic range. Among other capabilities, the tool allows manual curation to be performed and supports the gap-filling process of pathways that could not be determined entirely by name matching alone. This process relies on the gene sequences. ModelSEED (Devoid et al., 2013) is a web resource that facilitates the reconstruction, exploration, comparison, and analysis of organism-specific metabolic models. This tool relies on an initial genome annotation using RAST and the SEED ontology, clustering metabolic pathways into subsystems, which are further subclassified (Devoid et al., 2013). KEGG Mapper tools (Kanehisa and Sato, 2020) also allow automatic assignment of enzymatic roles and pathway contextualization using genomes or proteomes as input and relies on the KEGG ontology to perform annotations based on sequence similarity. Other tools, reviewed elsewhere, allow additional curation and pathway-specific analysis to be performed once a draft reconstruction is attained (Pitkänen et al., 2010; Abd Algfoor et al., 2017). Common to all described tools is their dependency upon a vocabulary of metabolic elements, or ontology, which depicts the complex, and often multi-level relationships among genes, proteins, enzymes, biochemical reactions, and regulators (Stobbe et al., 2012). Accordingly, metabolic reconstructions performed using different strategies may lead to differing outcomes for the same organism, as pathway representations and modeling varies among each developing group (Green and Karp, 2006).

Once a metabolic reconstruction is obtained for the studied pathogen, this compendium can be used during the early prioritization step that involves target identification, aiming to rank the proteins involved in critical metabolic roles or participate as key intermediaries of multiple pathways. This analysis can be facilitated using a graph representation of a metabolism (Ramos et al., 2018). Multiple topological criteria can then be employed to rank the proteins identified as belonging to one or more metabolic pathways (Box 1). The rationale behind this strategy is that drugs that inhibit such targets have higher chances of success than those that target non-essential cellular functions.

BOX 1 **Key concepts used to assign metabolic importance during target prioritization.**

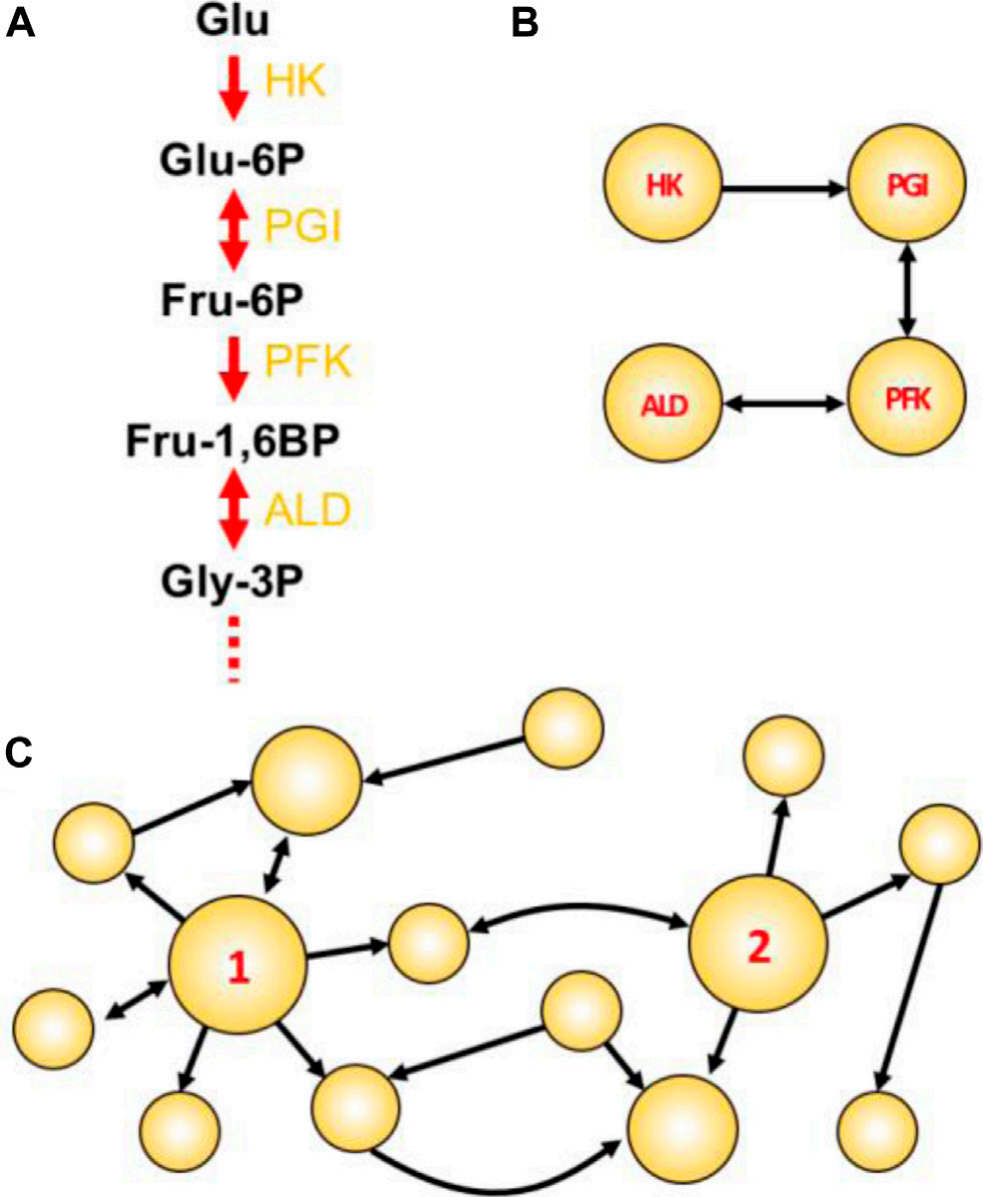

Figure Box Network concepts illustrated. **(A)** The initial four steps of glycolysis. In such traditional textbook representations, the emphasis is usually given to compounds. Enzyme names abbreviations are depicted in yellow. **(B)** A reaction-reaction directed graph constructed using the initial reactions shown in A. Here, the emphasis is given to reactions/enzymes. **(C)** A toy network with two particularly attractive nodes: node 1 having a high degree; and node 2 having high betweenness centrality. This example shows a directed graph, in which links are directed from one node to another. In undirected graphs, all links are bidirectional and represented by a single line connecting a node pair.
Choke-point reaction: A biochemical reaction that uniquely consumes (or synthesizes) a given substrate (or product) (Yeh et al., 2004). Enzymes that perform these reactions are termed choke-point enzymes, and their blocking could lead to the accumulation of the unique substrate (potentially toxic to the cell) or to the inability to produce an essential product (impairing the cellular homeostasis). For this reason, the identification of metabolic choke-points is integral to the prioritization of potential targets.
Reaction-reaction graph: In a graph-oriented study of metabolism (reviewed in (Cottret and Jourdan, 2010), biochemical reactions can be modeled as the network nodes, which consequently also model the enzyme(s) catalyzing the reaction. A link is placed between two reactions if one consumes a metabolite produced by the other reaction [Panel Box **(A,B)**].
Degree centrality (DC): The degree is one of many centrality measures (reviewed in (Jalili et al., 2016; Ashtiani et al., 2018) useful to define important metabolic nodes and represents the number of links connecting to a node. The higher the DC, the more shared metabolites a given reaction has with other immediate reactions. In directed graphs, the total DC is the sum of the in-degree (the number of incoming links) and out-degree (the number of outgoing links). An example of a high-degree node is shown in Panel Box **(C)**, where node 1 has in-degree = 2, out-degree = 6, and a total DC of 8.
Betweenness centrality (BC): Represents the frequency with which a given node appears as an intermediate between the paths of other possible node pairs. In the metabolic context, a reaction node with high BC would involve a metabolite that participates in many other reactions (not necessarily of the same direct pathway), thus having an important metabolic role. An example of a node having high BC is shown in Panel Box **(C)**, where node 2 is the only intermediate able to connect reactions on the left with the three reactions that appear on the right-hand part of the graph. Thus, node 2 is a key intermediate node in this graph.

### Target Selection Databases

The increased availability of pathogen genomes and genome-scale datasets are expected to guide target-based drug discovery projects. However, a major bottleneck has been the complexity of capturing and integrating relevant information available, making them accessible to experimental researchers, thus facilitating the identification and prioritization of potential antimicrobial targets. Nowadays, there are several freely available academic resources designed for antimicrobial target identification. Most of these tools focus on specific protein characteristics. For example, Drug Target Database is a useful resource to select potential targets based on a reverse docking approach. The Therapeutic Targets Database provides a large volume of data of already known therapeutic targets. Another database that includes data of known targets is TargetDB/TargetTrack (Chen et al., 2004), in spite of its focus on structural information.

There are also a few existing databases and resources aimed at a particular group of pathogens. TDR targets (Magariños et al., 2012) is an interesting tool focused on neglected tropical diseases. Regarding the prioritization of molecular targets in *M. tuberculosis*, two specialized databases are currently available, TuberQ and TargetTB. TuberQ provides a druggability analysis of the Mtb proteome contributing to a better selection of potential drug targets for screening campaigns (Radusky et al., 2014). TargetTB integrates network analysis of the protein-protein interaction, metabolism, essentiality, sequence analyses, and structural data (Raman et al., 2008). Some databases allow the use of metabolic network data to target prioritization, such as FindTargetsWEB. This web server takes as input an extended Systems Biology Markup Language (SBML) file of a metabolic model of the pathogen under study. It performs both flux balance analysis (FBA) and flux variability analysis (FVA) to prioritize bacterial molecular targets (Merigueti et al., 2019).

**FIGURE 1:**
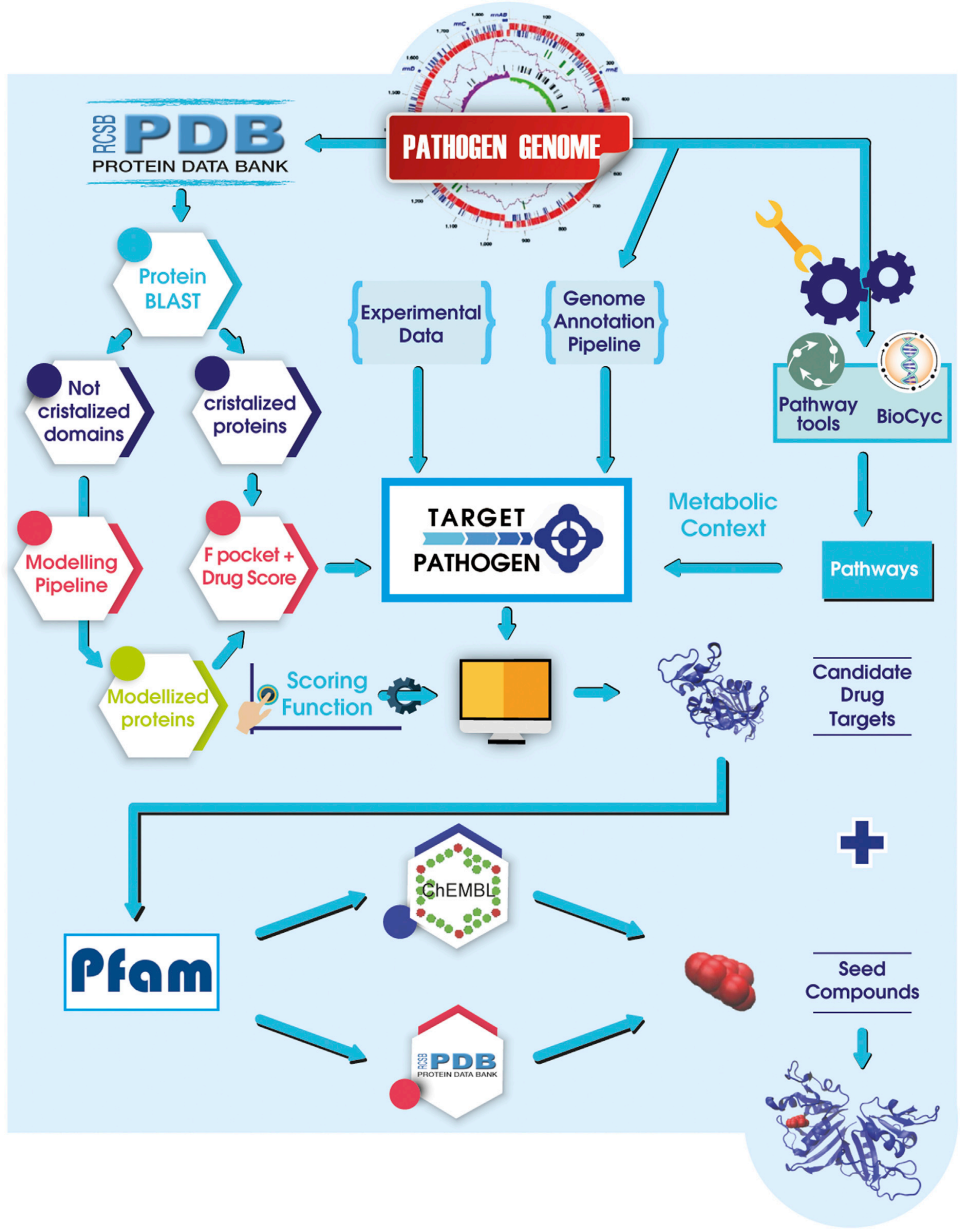
A general sketch of Target-Pathogen integrated with LigQ pipeline. Structural druggability and metabolic analyses are integrated with available experimental data and *in silico* analysis data. After all, data is integrated into Target-Pathogen, a user-designed scoring function is used to weight different features to obtain a ranked list of candidate drug targets. Once the target is selected, LigQ pipeline finds all known binders of similar proteins in the PDB and ChEMBL. Target and putative binders can be used in further molecular docking assays.

Most of the available user-friendly web servers use few data sources to prioritize targets. However, continuing efforts to allow target prioritization by applying integrated multi-data approaches are in ongoing focus. In this context, by combining structural druggability, essentiality analysis, metabolic context, as well as genomic and expression data, our group has developed Target-Pathogen (TP) (Sosa et al., 2018) (Figure 1). TP is a web server that enables to select and prioritize drug targets of several clinical pathogens, including *M. tuberculosis*, *M. leprae*, *K. pneumoniae*, *S. aureus*, *Schistosoma mansoni*, *Shigella dysenteriae*, *Toxoplasma gondii*, *Leishmania major*, *Trypanosoma cruzi*, *Acinetobacter baumannii*, and *Bartonella bacilliformis* among others. Under the TP framework, researchers can easily prioritize proteins of interest quickly and intuitively, running simple queries (such as searching for proteins with high druggability score or associated with metabolic reactions of high centrality), filtering by different data, assigning numerical weights for additional customized features and merge these results to obtain a ranked list of targets. A distinct advantage of the Target-Pathogen server is its capacity to rank, not solely proteins but entire pathways, thus allowing synergistically to attack several proteins of the same metabolic pathway. Another attractive feature of TP is that it will enable users to upload their data to be used in the prioritization pipeline. As of December 2020, there are 25 of the most relevant microorganisms from the human health perspective. Users can also request new genomes to be included in the platform by emailing target@biargentina.com.ar. By abiding to open-science practices, data associated with protein structures can be downloaded to perform further *in silico* analysis outside TP.

### Identifying Lead Compounds to Treat Bacterial Infections

Once the protein target is selected, the challenge moves from biology to chemistry and consists of the identification of a small, usually drug-like, molecule that can inhibit the target’s function, allows further pharmacological validation of the target, and ultimately paves the way for the development of a new antibiotic. To test a molecule’s capacity to inhibit the desired target, *in vitro* protein activity (or binding) assays can be performed, as well as cell culture MIC determinations. However, the problem is that the universe of molecules that could act as inhibitors is vast. Conducting experimental high-throughput screening is beyond the capacity of most academic research labs in Latinamerican countries, where a typical research group can afford and test about 100 compounds in a typical one by one *in vitro* assay each year. Therefore, usually, only a moderate number of compounds are tested, and bioinformatics methods capable of screening for potential binders are highly appreciated.

The computational selection of potential inhibitors against a defined target is generally referred to as Virtual Screening (VS). VS methodologies can be further divided into two main techniques, which can be applied sequentially to obtain a best set of potential inhibitors. The first relies mainly on previous biological information and chemical similarity analysis of the compounds. It is usually referred to as compound filtering or pre-selection (as will be described below). The second, which is computationally demanding, involves molecular docking of each compound in the protein target, estimating its binding free energy, and finally performing a ranking. This technique is commonly and traditionally referred to as VS in the strictest sense.

Compound filtering traditionally involves selecting drug-like compounds using a set of driving principles, for instance, Lipinski's rules (Lipinski et al., 2001). However, the increasing amount of information available in public databases allows the derivation of improved filters, e.g., using the “guilt by association” principle, as described in our previous work LigQ (Radusky et al., 2017), and similar developments (O’Boyle et al., 2011; Volkamer et al., 2012). The idea is that similar proteins bind similar compounds. Therefore, for a given target, those compounds that are similar (in chemo-structural properties) to known binders of similar (homolog) proteins are good candidates. Starting from the selected target (protein name or UniprotId), LigQ first finds all known binders of similar proteins. Binders are classified in groups according to the degree of protein similarity [starting from high identity >60% homologs to binders to the same domain in PFAM (Mistry et al., 2020)] and available information (such as the structure of the protein-ligand complex) in different databases such as Protein Data Bank (PDB; http://rcsb.org), Pfam (http://pfam.xfam.org/), and ChEMBL (EMBL-EBI; http://www.ebi.ac.uk/chembl/).

This set of compounds is called the “seed set.” It is used to retrieve from large datasets of commercially available compounds, those that are chemically similar to a specific -user-defined- degree. Chemical similarity can be defined based on the Tanimoto Index (Bajusz et al., 2015), and the similarity retrieval cut-off can be used to select the number of compounds to be retrieved, which are also organized in clusters according to their chemical similarity.

The information is extracted for each database, constituting four individual seed sets (Seed I–IV). Seed I and III are obtained through the direct search of the protein of interest by its corresponding identifier (ID) for each base (PDB (Sussman et al., 1998) and ChEMBL (Gaulton et al., 2012), respectively). On the other hand, the seeds II and IV are extracted by previously obtaining the functional domains (Pfam) that compose the protein of interest by using HMMER and later searching in PDB and ChEMBL for the compounds that interact with these domains.

In the following sections, we present and review prioritized targets and their potential binders, identified using the above-described methodology for two bacterial pathogens with an important impact in Latin America: *Bartonella bacilliformis* (causal agent of Carrion’s disease) and *Mycobacterium tuberculosis*.

## *Bartonella bacilliformis* and Carrion’s Disease

Carrion’s disease is an ancient vector-borne biphasic illness dating from the pre-Columbian era, restricted to the South American Andes, including Peru, Ecuador, and Colombia (Gomes and Ruiz, 2017). It is an endemic illness found in Andean valleys at an altitude of 600–3,200 m above sea level. *B. bacilliformis* (Bb) is transmitted to humans by female sandflies belonging to the *Lutzomyia* genus, which are commonly present in Andean valleys’ high-altitude regions (Clemente et al., 2012; Minnick et al., 2014). However, since the end of the last century, an expansion of the illness into bare areas including jungle and coastal regions, such as the coastal areas of Guayas and Manabi in Ecuador, has been reported (Gomes and Ruiz, 2017; Garcia-Quintanilla et al., 2019). It is also thought that climate change could favor the expansion of Bb infections, presumably affecting the vector proliferation. In this sense, it is worthwhile to mention the El niño phenomenon, the unusual warming of surface waters in the eastern Pacific Ocean, which leads to a temperature and humidity increasing. These climate characteristics especially favor the sandfly spreading and promoting new Carrion’s disease outbreaks (Pons et al., 2016).

The infection caused by Bb has two well defined clinical phases. The early stage, denominated Oroya fever, causes a severe acute hemolytic anemia. High case-fatality rates as 40–88% have been described in the Oroya fever phase in patients without any antibiotic treatment. Even with timely antibiotic treatment, the fatality rate is around 11% (Farfán-López et al., 2020). The chronic phase of Carrion’s disease is characterized by the development of dermal eruptions known as Peruvian warts and commonly present on the head and extremities. Although this phase is seldom fatal, dermal eruptions can be accompanied by fever, headache, lymphadenopathy, and acute pains in joints and bones (Minnick et al., 2014).

Regarding antimicrobial therapy to treat Carrion’s disease, different antibacterial agents have been used since the beginning of the antibiotic era, such as beta-lactams (including penicillins and cephalosporins), aminoglycosides, and quinolones (Battisti et al., 1998). Although most Bb strains are sensitive to a broad set of antimicrobials *in vitro*, there is still a potential risk of developing antibiotic-resistance during clinical treatment. Oroya fever has been traditionally treated with chloramphenicol, a successful drug due to the frequent coinfection with *Salmonella* spp. However, it is nowadays restricted for humans because of its potential to produce side effects in the bone marrow. Other drugs to treat Carrion’s disease include beta-lactams such as ampicillin and penicillin G, tetracyclines (doxycycline), macrolides (erythromycin, roxithromycin), trimethoprim-sulfamethoxazole, and fluoroquinolones (norfloxacin, ciprofloxacin) (Rolain et al., 2004). Although the second-generation fluoroquinolone ciprofloxacin is the drug of choice for treating acute cases, it should be judiciously recommended because of the ability of Bb to become quinolone resistant. Several studies showed that quinolone resistance-determining regions (QRDR) are consequences of synonymous or non-synonymous mutations and responsible for the intrinsic resistance of *Bartonella* spp to this antimicrobial (Valle et al., 2010); (Espinoza-Culupú et al., 2014). Additionally, mutations conferring resistance to ciprofloxacin, erythromycin, rifampin, aminoglycosides, and folate inhibitor targets have been molecularly characterized in clinical isolates (Biswas et al., 2007). The current scenario is worse, considering the antibiotic resistance mediated by efflux pump overexpression (Gomes et al., 2016).

### Mycobacterium Tuberculosis

Tuberculosis (TB) is an infectious disease that accounted for 1.2 million deaths in 2019 (Harding, 2020) being one of the top ten causes of death worldwide and the leading cause of death from a single infectious agent (ranking above HIV/AIDS). More than 95% of cases and deaths occur in developing countries (Ascenzi and Visca, 2008). Tuberculosis epidemiology varies markedly between Latin American countries (Woodman et al., 2019). The incidence of tuberculosis in Central America (including Mexico), the Caribbean, and South America were 25.9, 46.2, and 61.2 per 100,000 people. Drug resistance is an increasing problem throughout the Americas, particularly in Peru, where drug-resistant tuberculosis accounts for 9% of the cases (Woodman et al., 2019). In this framework, only 33% of patients received drug-susceptibility testing, resulting in an estimated 7,000 undiagnosed or untreated patients with drug-resistant tuberculosis (Woodman et al., 2019). About a quarter of the Latin American population is latently infected with Mtb.

Immune response to Mtb relies on phagocytosis of the bacteria by macrophages leading to granuloma formation. Inside the macrophages, bacilli face stressful conditions characterized by the presence of Reactive Nitrogen and Oxygen Species (RNOS). Based on this observation, we have hypothesized that identifying Mtb RNOS protein targets would permit us to select inhibitors against them and synergize with the macrophages attack in the latent phase of the infection (Defelipe et al., 2016).

### Exploring the Druggable Genomes of *Bartonella bacilliformis* and *Mycobacterium tuberculosis*

**FIGURE 2:**
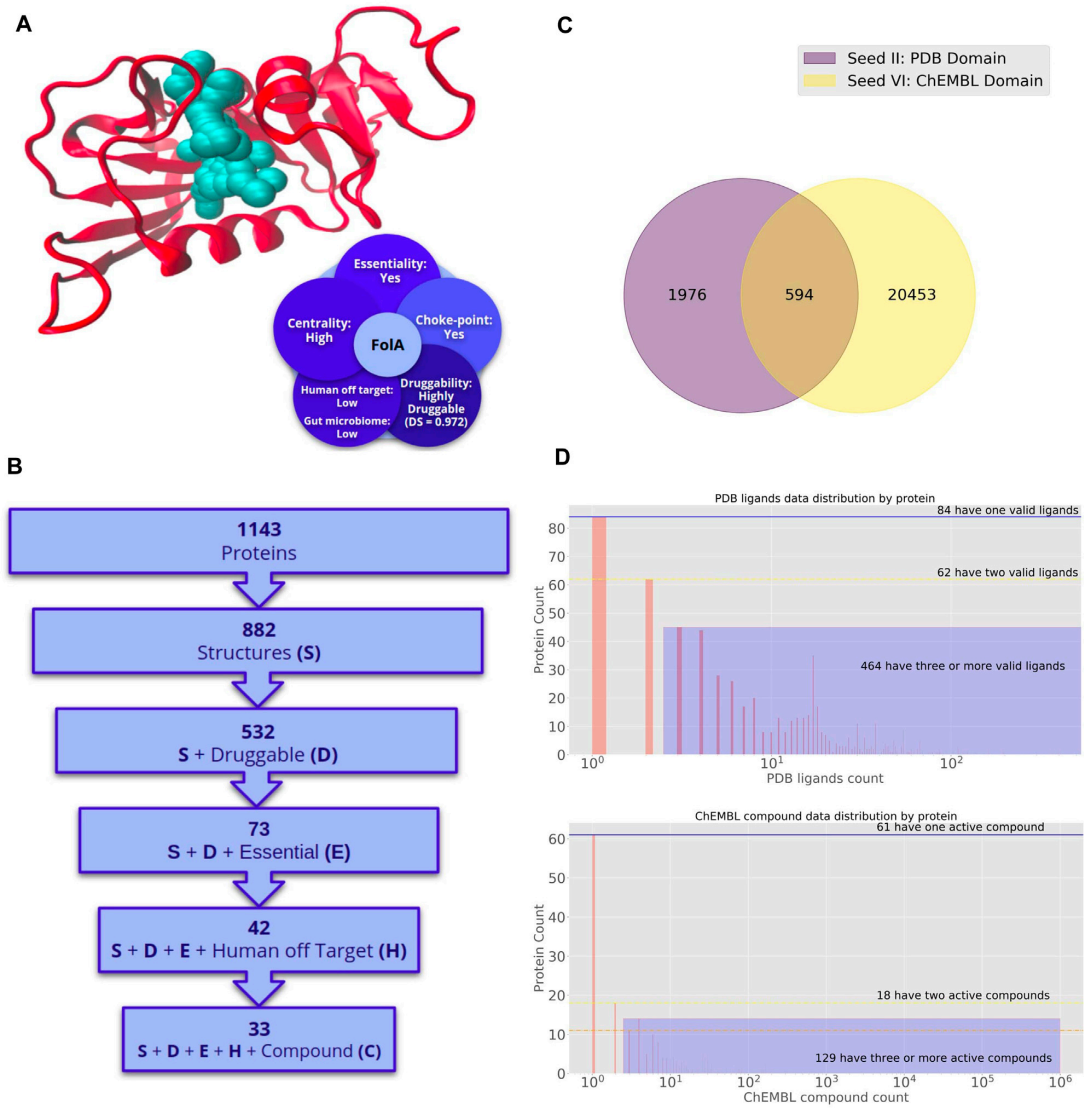
Potential druggable targets and putative lead compounds to combat *Bartonella bacilliformis*. **(A)** Dihydrofolate reductase (FolA) structure. Crystallographic structure and a set of attractive features for target prioritization are shown. **(B)** Number of proteins in *Bartonella bacilliformis* genome with desirable properties for drug targets. Different filters are sequentially applied to obtain a shortlist of druggable, essential, and low identities with proteins in the human genome. The last filter is applied to get the list of proteins with putative binders. **(C)** Seed Compounds for *Bartonella bacilliformis*. Venn diagram of the number of seed compounds corresponding to different sets. Binders are classified into two seed groups. Seed II are those drugs that bind any protein that harbor the same Pfam domains with Bb proteins and have been co-crystallized with such proteins in the PDB. Seed IV is the set of drugs that bind any protein that shares Pfam domains with *Bartonella bacilliformis* proteins and was reported as active in Chembl. **(D)** Number of proteins in the *Bartonella bacilliformis* genome for which a ligand can be predicted. The top panel corresponds to drugs in Seed II. The bottom panel corresponds to drugs in Seed IV.

We applied the previously described pipeline to the pathogens mentioned above, Bb and Mtb. Below we present the application of successive filters (drugability, essentiality, etc.) along their genomes. Bb genome codes for 1,143 different proteins (Figure 2B), from which we were able to build a total of 882 high-quality structural models (no experimental structures are available in the PDB for any Bb protein). Homology-based models are built for all proteome sequences using MODELLER (Webb and Sali, 2016) only when an adequate template is available (coverage 80%, E-value > 1 × 10^5^). Only those models with GA341 score above 0.7, QMEAN between −2 and two are retained. It has been shown that the RMSD between the Modeller models and the native structures is <3 Å (Wallner and Elofsson, 2005), which shows the quality of the obtained models. 532 (∼60%) of the models harbored a druggable pocket (DS > 0.5). From this subset of structurally relevant proteins, only 73 can be predicted as essential (i.e., close homologs in the Database of Essential Genes were found). After further filtering those proteins with close homologs in the human genome, 42 proteins remained (identity < 0.4). When an additional filter was applied in TP to disclose proteins that could potentially bind at least one compound, a final set of 33 candidate proteins was obtained.

Additionally, 18 of the 42 proteins mentioned above also have a low impact on the gut microbiome, and 17 have putative binders. Ten targets (all with predicted ligands) are also associated with choke-points reactions, and four with high-centrality reactions from the metabolic network point of view (concepts defined in Box 1). All this information is provided in the TP database, while a detailed list of mentioned targets is also presented in Supplementary Table S1.

**FIGURE 3:**
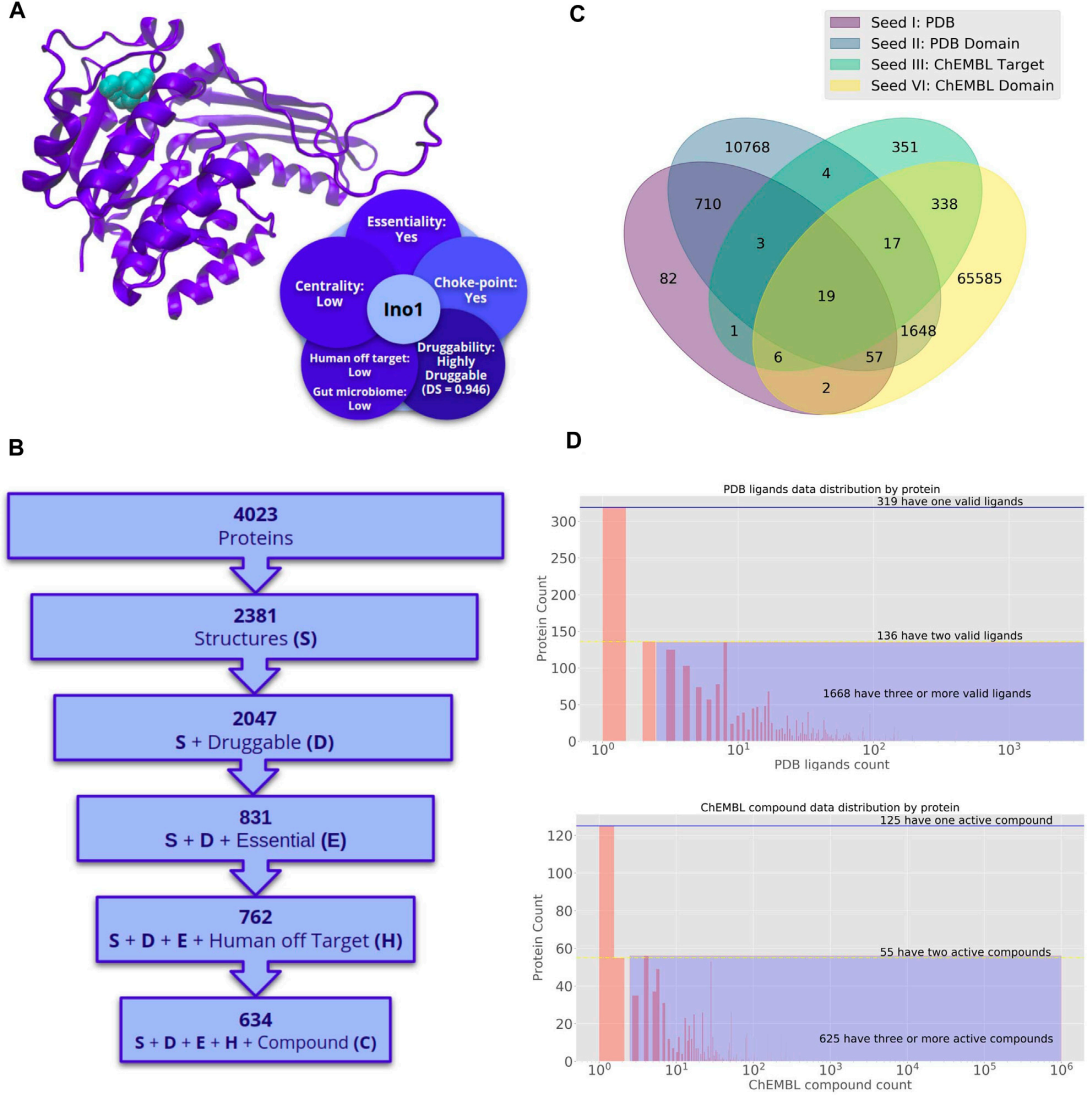
Potential druggable targets and putative lead compounds to combat *Mycobacterium tuberculosis*. **(A)** Inositol-3-phosphate synthase (Ino1) structure. Crystallographic structure and a set of attractive features for target prioritization are shown. **(B)** Number of proteins in *Mycobacterium tuberculosis* genome with desirable properties for drug targets. Different filters are sequentially applied to obtain a shortlist of druggable, essential, and low identities with proteins in the human genome. The last filter is applied to get the list of proteins with putative binders **(C)** Seed Compounds for *Mycobacterium tuberculosis*. Venn diagram of the number of seed compounds corresponding to different sets. Binders are classified in groups according to the degree of protein similarity [starting from high identity >60% homologs to binders to the same domain in PFAM (Mistry et al., 2020)] and available information (such as the structure of the protein-ligand complex) in different databases such as Protein Data Bank (PDB), Pfam, ChEMBL (EMBL-EBI). Venn diagram of the number of seed compounds corresponding to different sets. Binders are classified into two seed groups. Seed I and III are obtained through the direct search of the protein of interest by its corresponding identifier (ID) for each base and ChEMBL. Seed II are those drugs that bind any protein that harbor the same Pfam domains with Bb proteins and have been co-crystallized with such proteins in the PDB. Seed IV is the set of drugs that bind any protein that shares Pfam domains with *Mycobacterium proteins* and was reported as active in Chembl. **(D)** Number of proteins in the *Mycobacterium tuberculosis* genome for which a ligand can be predicted. The top panel corresponds to drugs in Seed II. The bottom panel corresponds to drugs in Seed IV.

The Mtb proteome comprises 4,023 proteins (Figure 3B), being 2,381 structurally defined (382 experimental structures and 1,999 models). From these proteins, 2,047 had DS > 0.5 (∼85%), 831 were also essential, and putative drugs delivered for 762 are predicted to have a low impact in humans. We could predict possible binders for 634 proteins out of this 762. From this subset, 635 satisfy microbiome off-target criteria (527 has possible binding compounds). If metabolic perspective is also considered, 140 catalyze choke-points reactions (130 with potential binders), and seven are associated with-high centrality reactions (all with predicted ligands). All this information is provided in the TP database, while a detailed list of mentioned targets is also presented in Supplementary Table S2.

The successive steps of the pipeline indicate how the application of sequential filtering steps narrows the universe of potential targets. We describe the most promising targets and their potential inhibitors for Bb and Mtb in the following sections.

### Bb Prioritized Protein Targets and Their Potential Inhibitors

In Farfán-López et al. (2020), our group participated in a work that combined the efforts of scientific groups from Argentina, Brazil, and Peru to perform an integrative genomic-scale data analysis, which allowed us to shortlist a set of proteins that could serve as attractive targets for new antimicrobial discovery projects against Bb. This study was based on the genomic analysis of Bb USM-LMMB 07, firstly isolated in 2011 during an outbreak in Carmen de la Frontera district, Huancabamba Province, Piura (Guillen et al., 2016). The combination of genomic, structural, metabolic, and functional data integrated inside Target-Pathogen, finally led to shortlisting six proteins (FabI, FolA, AroA, TrmFO, UppP, and MurE) with unique characteristics (Table 1). FolA provides the main dihydrofolate reductase activity in the tetrahydrofolate or vitamin B9 pathway (Figure 2A). As is well known, tetrahydrofolate is a crucial intermediate in the biosynthesis of nucleic acids and proteins, which is biosynthesized *de novo* in bacteria. It participates in essential biosynthesis pathways, such as methionine, purines, and thymidylate. Since dihydrofolate reductase is essential for cell division and growth, it could become an attractive target for drug development. Another top-ranking protein is Enoyl- [acyl-carrier-protein] reductase (FabI), which is involved in fatty acid biosynthesis processes and was also described to be essential in many other bacteria, such as *E. coli* and Mtb (Heath et al., 1998; Kaplan et al., 2012). The gene product of *aroA* also meets the standard requirements to become a potential molecular target. Our ontology analysis results revealed that this protein is involved in aromatic amino acids and chorismate biosynthesis and showed an essential role in *Rhodopseudomonas palustris* CGA009 and *Caulobacter crescentus* (53.3 and 50.9% sequence identity with Bb *aroA,* respectively). Another attractive target found by our subtractive genomic approach is the tRNA modification enzyme, TrmFO. This protein showed a high identity against the *gid* essential gene of *Staphylococcus aureus* N315. Interestingly, several pathogens such as *E. coli*, *P. aeruginosa*, and *S. enterica* (Yim et al., 2006; Gupta et al., 2009; Shippy et al., 2011) show pleiotropic effects when carrying a mutant *gidA*; thus in this line, TrmFO becomes an appealing target in Bb. Finally, we prioritized UppP and MurE, enzymes involved in peptidoglycan (PG) biosynthesis, usually considered one of the principal antimicrobial targets. PG is a crucial component of the cell envelope of Eubacteria. It has an essential role in bacterial physiology due to its functions in maintaining the shape and integrity during growth and cell division, controlling the internal turgor pressure resistance, and serving as a structural scaffold to other cell envelope components. We now turn our attention to their potential inhibitors.

**TABLE 1:** Proteins of Bb with desirable features to become a promising drug target.

*Bartonella bacilliformis*						
Protein name	Druggability	Choke point	Centrality	Human off-target	Gut microbiome	Essentiality
Enoyl-[acyl-carrier-protein]	0,992	Yes	High	Low	Low	Yes
Reductase (FabI)						
Dihydrofolate reductase (FolA)	0,972	Yes	High	Low	Low	Yes
3-Phosphoshikimate carboxyvinyltransferase (AroA)	0,775	Yes	High	Low	Low	Yes
FADH(2)-oxidising methylenetetrahydrofolate	0,746	Yes	High	Low	Low	Yes
–tRNA-(uracil(54)-C(5))- methyltransferase						
(TrmFO)						
Undecaprenyl-diphosphatase (UppP)	0,738	Yes	High	Low	Low	Yes
UDP-N-acetylmuramoyl-L-alanyl-D-glutamate--2,6-diaminopimelate ligase (MurE)	0,952	Yes	High	Low	Low	Yes

As described in the introduction, we implemented the LigQ pipeline in the context of Target-Pathogen to allow the identification of potential ligands that interact with desired protein targets. As mentioned above, there are no Bb protein structures in the PDB. There is also no information on experimental assays in ChEMBL for this pathogen; therefore, the set of possible inhibitors is based on seed sets II and IV, i.e., derived from ligands observed for proteins that share domains with the selected Bb targets. Seed set II consisted of 1,976 compounds, while seed IV was composed of 20,453 drugs. In contrast, 594 compounds are retrieved from both PDB and ChEMBL simultaneously (Figure 2C). This fact makes these drug-like compounds attractive to combat Bb infections. From a total of 1,143 Bb proteins, we could predict possible binders for 610 in the PDB and 201 in ChEMBL (Figure 2D). Potential inhibitors for the predicted targets are shown in Supplementary Table S3. As an example, Isoniazid (CHEMBL64—INH) was indicated as a potential inhibitor of Bb FabI. INH is one of the most important first-line drugs against tuberculosis. Although antimicrobial activity of INH is thought to be selective for mycobacteria, likely due to its ability to inhibit mycolic acid synthesis, Bb FabI and Mtb InhA (the protein target of INH) share the same and domains and are structural homologs, except for the presence of a long loop of interaction with the substrate found in InhA (Andrade et al., 2008). Moreover, it was recently shown that isoniazid in conjugation with nanoparticles could prevent the growth of *Enterococcus faecalis, E. coli, Pseudomonas aeruginosa,* and *S. aureus* (Zargarnezhad et al., 2020). This prodrug is activated by the heme enzyme catalase-peroxidase (KatG) endogenous to *M. tuberculosis.* Given this information, it is possible to propose INH, or its Mtb endogenous product (after reaction with KatG), as a potential compound for future trials against Bb.

Another interesting compound found was Fosmidomycin (CHEMBL203125). This compound has recently completed the clinical phase III for *Plasmodium* infections, although its mechanism of action is not entirely understood. It is reported that this compound is active against UDP-N-acetylglucosamine 1-carboxyvinyltransferase (MurA) in *E. coli*. MurA shares the same Pfam domain (PF00275) as Bb. In this way, we can think of Fosmidomycin as an attractive seed compound to be used in drug discovery projects against this bacteria.

### *Mycobacterium tuberculosis* Prioritized Targets and Their Potential Inhibitors

To further analyze the potential of the 743 Mtb proteins, which are essential and druggable, an analysis of available expression data under different infection mimicking conditions was previously performed (Starvation, Hypoxia, RNOS stress, and mice infection) (Defelipe et al., 2016). We found that 24 of these proteins were also overexpressed in at least three conditions, including DevS protein, known to be involved in RNOS sensing and signal transduction, harboring a druggable kinase ATP binding pocket.

As a last step in the prioritization procedure, a comprehensive Mtb metabolic network was built. As mentioned above, Target-Pathogen allowed us to score not individual proteins but entire pathways, according to their potential to be used as targets in latent tuberculosis drug discovery projects. In this framework, all pathways that do not have at least one druggable protein were ruled out, and a scoring function was developed to combine each protein data into a global network score.

This analysis revealed several high-scoring “druggable” pathways, which include a set of targets with great potential for further drug discovery projects (Table 2). One of them was the mycothiol biosynthesis pathway. Mycothiol is crucial for the intracellular redox balance and plays a crucial role in Mtb survival within macrophages (Buchmeier et al., 2003). Inositol-3-phosphate synthase (Ino1, Rv0046c), an enzyme involved in the early steps of this pathway, is highly druggable and over-expressed in RNOS stress, hypoxia, and starvation, three of the four latent infection mimicking conditions (Figure 3A). Mycolate biosynthesis pathway is also at the top of the ranking. Mycolate is an integral cell wall component of Mtb that participates in the survival ability of the bacilli within infected hosts, virulence, and evasion of the immune system. This pathway is targeted by first-line tuberculosis drugs such as isoniazid and ethambutol (Barry et al., 2007) and harbors the promising target 3-oxoacyl-[acyl-carrier protein] synthase 2 (KasB, Rv2246) involved in meromycolate extension. The scoring function also reveals the relevance of sulfur metabolism, essential for the bacilli’s survival and virulence. Moreover, most genes are absent in humans. Among these pathways, methionine degradation to homocysteine is performed by the druggable protein Rv3340 (O-acetylhomoserine amino carboxypropyl transferase), another interesting target for future developments. Chorismate biosynthesis was another prioritized pathway. Chorismate is a key biochemical intermediate, being a precursor for aromatic amino acids. Within this pathway, 3-phosphoshikimate 1-carboxyvinyltransferase (Rv3227) could be selected for further studies. We found that it is druggable, essential, and not present in humans, and appears overexpressed under different conditions that mimic infections.

**TABLE 2:** Mtb proteins with worthy properties that make them good candidate targets.

*Mycobacterium tuberculosis H37Rv*						
Protein name	Druggability	Choke point	Centrality	Human off-target	Gut microbiome	Essentiality
Inositol-3-phosphate synthase (Ino1, Rv0046c)	0,946	Yes	Low	Low	Low	Yes
3-Phosphoshikimate 1-carboxyvinyltransferase (Rv3227)	0,696	Yes	High	Low	Low	Yes
O-Acetylhomoserine aminocarboxypropyltransferase (Rv3340)	0,679	Yes	Low	Low	High	Yes
3-Oxoacyl-[acyl-carrier-protein] synthase 2 (Rv2246)	0,709	Yes	Low	Low	Low	Yes
Octanoyltransferase (Rv2217	0,703	Yes	Low	Low	Low	Yes
Bifunctional protein GlmU (Rv1018c)	0,833	Yes	High	Low	Low	Yes
Rv1465	0,802	Yes	Low	Low	Low	Yes

Among other top-scoring pathways revealed by our analysis are those related to lipoate synthesis. The two key genes (*lipA*, Rv2218 and *lipB*, and Rv2217) are essential, and *lipB* was also found to be druggable and expressed under starvation conditions. Moreover, the druggable pocket of LipB has Cys 176, Tyr22, and Tyr 91, making the pocket potentially sensitive to RNOS. Although this process is not ubiquitous in Bacteria, lipoate has been implicated in microbial pathogenesis, including immune response-induced oxidative and nitrosative stress in mycobacteria. It has also been acknowledged that lipoylated proteins take part in crucial antioxidant processes (Spalding and Prigge, 2010), thus promoting this high-scoring pathway from the target-finding aspect. Moreover, LipB has been structurally characterized and shown to have promising therapeutic properties (Ma et al., 2006). Other worth mentioning pathways are the UDP-N-acetyl-D-glucosamine biosynthesis I and iron-sulfur cluster biosynthesis, which harbors attractive targets, such as Rv1018c (GlmU) and Rv1465 that have a set of desirable characteristics to be considered as promising targets to combat latent tuberculosis (Table 2). Regarding possible binders for Mtb proteome, 82, 10,768, 351, and 65,585 compounds make up seed I, II, III, IV sets, respectively. Whereas 19 ligands are obtained in the four seeds in parallel, which make them attractive compounds to treat Mtb infections (Figure 3C). Concerning the distribution of ligands by proteins (Figure 3D), 2,123 Mtb proteins interact with at least one compound, in the case of PDB (Figure 3D, top), 319 interact with a single ligand, 136 with two, and 1,668 with at least three (Purple shading). In the case of ChEMBL (Figure 3D, bottom), 125 interact with a single ligand, 55 with two, and 625 with at least three (purple shading). We could predict ligands for 2,123 Mtb proteins by searching in the PDB and 805 by looking in ChEMBL. The putative inhibitors found for the targets mentioned above are summarized in Supplementary Table S4. Worth to mention is Disulfiram (CHEMBL964) that was found to target GlmU. This drug inhibits enzymatic oxidation and is widely used to support the treatment of chronic alcoholism, different types of cancer, and parasitic infections. Furthermore, it has recently been proposed as an antibacterial compound against methicillin-resistant *S. aureus* (MRSA) and vancomycin-resistant *Enterococcus* (VRE) (Frazier et al., 2019) and particularly Mtb (Horita et al., 2012; Chaudhary et al., 2020).

## Discussion

In the last decades, antimicrobial drug development has observed a shift from the traditional approaches based mostly on phenotypic screening of natural/synthetic compounds to a rational genome-based target-driven lead discovery approach. Since wet-lab investigations of candidate targets and lead compounds are time-consuming and expensive, it is worthwhile to conduct bioinformatic analyses to identify the proteins and ligands most worthy of experimental follow-up. *In silico* analyses are particularly important in developing countries (such as those from Latin America), where the research investment is usually limited.

Our developed bioinformatics pipeline and the underlying methodology, briefly presented here for Bb and Mtb, and freely available to the scientific community at http://target.sbg.qb.fcen.uba.ar/patho/, allows starting from a pathogen whole genome, the modeling and classification of the proteome. General results show that a large fraction of protein structures harbor a druggable pocket (60–85%). Interestingly, effects on the essential proteins yield substantial differences between both bacterial pathogens. While 20% of Mtb druggable proteins were considered essential, only 6% of the Bb proteome resulted in druggable and essential proteins. These differences could be explained by the different amounts of data available, particularly in terms of essentiality and knowledge on gene/protein function for both microorganisms, Mtb and Bb. Although there is vast information available for Mtb*,* Bb is a neglected disease with only a regional impact. Specifically, Mtb essentiality criteria were based on experimental mutagenesis studies; meanwhile, Bb essential genes were inferred by homology analysis with the Database of Essential Genes.

The results presented here (further expanded in the web Target-Pathogen) provide two crucial assets for those researchers in the field of Bb or Mtb antimicrobial development. In the first place, we provide a shortlist of attractive protein targets in each pathogen (Tables 1, 2). We also provide a detailed analysis of those characteristics that make it a good target for each gene/protein (essentiality, druggability, biological relevant role, and lack of cross-reactivity with the host). We hope this analysis will allow wet-lab researchers to develop upon the targets disclosed herein, moving research forward. The second, and more important issue, is that we provide a list of potential inhibitors and their chemical scaffolds for several prioritized targets. (Supplementary Tables S3, S4). We expect that researchers working with those targets and familiar with whole-cell and protein-based *in vitro* will become interested and directly try some of these compounds for their antimicrobial activity. Furthermore, the current pipeline is also presented for other targets and pathogens in our freely accessible website, thus providing the community with a general platform to drive the development of antimicrobial compounds forward.

*In silico* approaches are rapid, efficient, and cost-effective techniques for screening drug targets and narrowing the search space of drug like-compounds for any given pathogen. The goal of these techniques is not to replace wet-lab strategies. Instead, it is to become a useful resource for researchers working in target identification and drug discovery to translate biological questions in a computationally tractable way by filtering and weighting the vast quantity of genome-scale data sets. High-throughput screening (HTS) campaigns against molecular targets *in vitro*, although extremely valuable, typically do not yield directly good antimicrobial compounds (Payne et al., 2007; Tommasi et al., 2015). The bacterial cell envelope has evolved to refract toxic compounds from entering into the cell and even those drugs that cross the barrier, can be extruded by efflux pumps in multidrug resistant bugs (Li et al., 2015), thus resulting in poor *in vivo* activity. Another limitation of HTS approaches, is that only a finite amount of chemicals, with limited diversity, are available in any given library. As this chemical space limitation will hardly be overcome, novel approaches are needed to tackle the ongoing problem of bacterial resistance to current treatments. Our work provides a framework for which such novel strategies can be developed and further adapted to use by mean sized research laboratories including those of developing countries. Our strategy looks first for potential best molecular targets, and subsequently applies *in silico* screening to find best drug candidates. Novel methods for drug delivery, in particular nanomaterials and molecular transporters have started to be investigated as alternative antibacterials or anti-infective carrier systems to improve the internalization of bactericidal drugs against bacterial infections, which are particularly problematic in the case of having to reach the cytoplasm, specially in Gram-negative pathogens. Some of these promising molecules that could help overcome the bacterial envelopes and are currently being tested are siderophores, cyclodextrins, and metal nanoparticles, antimicrobial/cell-penetrating peptides and fusogenic liposomes (Santos et al., 2018). In this sense, we believe in a strategy that combines omics data and drug screening to discover lead antimicrobials, in which *in silico* and wet-lab approaches act synergically to maximize the success rate of drug discovery projects.
